# Alpha‐thalassemia. Case report alpha‐thalassemia in a Costa Rican family, A case report

**DOI:** 10.1002/ccr3.3518

**Published:** 2020-11-29

**Authors:** Mariela Solano‐Vargas, David H. K. Chui, Walter Rodriguez‐Romero

**Affiliations:** ^1^ Research Center of Hematology and Related Disorders (CIHATA) Universidad de Costa Rica San José Costa Rica; ^2^ Hemoglobin Diagnostic Reference Laboratory Boston Medical Center Boston MA USA

**Keywords:** ( – – ^SEA^/) deletion, alpha‐thalassemia, capillary electrophoresis, Chinese ethnic, Gap‐PCR

## Abstract

This case report highlights the importance for health care providers to be aware of the αlpha‐thalassemia syndromes, their relevance to clinical care and family counseling, appropriate diagnostic algorithm for definitive diagnosis.

## Graphical abstract

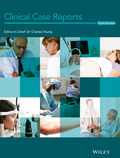

## 1 BACKGROUND

The ( – – ^SEA^/) deletion is very common in southern China and in southeast Asia. We report a Costa Rican family of Chinese ethnic background (Guangdong province) with this mutation. This report emphasizes the importance of the alpha‐thalassemia syndromes, their carrier prevalence in different populations, relevance in clinical care and family counseling, definitive diagnosis, and distinction from iron deficiency.

There are more than 1800 known mutations/deletions/insertions/rearrangements affecting human globin genes, including more than 800 involving α‐globin genes.^1^ Deletions of single α‐globin gene of the rightward and leftward types are common and found in all populations. People who are carriers of either deletion are well and do not have significant hematological findings. However, in combination with a deletion of both α‐globin genes in cis, the affected individual will have HbH disease which can present with clinically significant anemia.^2^

There are more than 20 known large deletions that remove both α‐globin genes in cis. All of them are rare, with one exception. The southeast Asian type of α‐globin gene deletion ( – – ^SEA^/) of ~20.5 kb in length, deleting both α‐globin genes in cis but sparing the embryonic ζ‐globin gene, is very common in southern China and in southeast Asia. The carrier frequency of this deletion varies from 4.5% in Hong Kong to as high as 14% in northern Thailand. Heterozygotes of this deletion are clinically well, but have mild anemia with significant microcytosis and hypochromia. Affected individuals might be mistaken for being iron deficient and thus given iron supplement inappropriately. Importantly, depending upon the α‐globin genotypes of their partners, offsprings of these carriers might have HbH disease, or the devastating Hb Barts hydrops fetalis syndrome.^3^, ^4^

## 2 CASE PRESENTATION

A 35‐year‐old Costa Rican woman of Chinese ethnic background (Guangdong province) presented with near normal Hb (11.2 g/dL) but significant microcytosis (MCV 64.9 fL) and hypochromia (MCH 20.6 pg).

## 3 METHODS

Hemoglobin analysis by capillary electrophoresis (Minicap Flex Piercing) revealed normal Hb pattern with HbA_2_ at the lower limit of normal. For DNA‐based α‐globin genotyping, we initially carried out reverse‐hybridization for α‐thalassemia (ViennaLab StripAssays^®^).^5^

## 4 RESULTS

A family study of both her parents and younger brother was undertaken (Table 1). Her father and her brother also have mild anemia with microcytosis and hypochromia. Treatment with brilliant cresyl blue yielded inclusion bodies in erythrocytes from the proband, her father and her brother.

**TABLE 1:** Hematological parameters in α‐thalassemia family case

Parameters	Father	Mother	Son	Daughter
Age (years)	74	69	34	35
Hb (g/dL)	9.8	10.7	11.3	11.2
MCV (fL)	69	89.4	62.7	64.9
MCH (Pg)	22.5	29.2	20.1	20.6
HbA (%)	98.0	97.3	97.8	98.0
HbA_2_ (%)	2.0	2.7	2.2	2.0
RBC (x10^6^/uL)	4.36	3.67	5.63	5.44
Inclusion bodies	Positive	Negative	Positive	Positive
Blood smear	Microcytosis ++, hypochromia+	Normocytic normochromic	Microcytosis ++, hypochromia++	Microcytosis ++, hypochromia+
α Genotype	‐‐SEA/αα	αα/αα	‐‐SEA/αα	‐‐SEA/αα

Abbreviations: Hb, hemoglobin; MCH, mean corpuscular hemoglobin; MCV, mean corpuscular volume; RBC, red blood cell count.

The proband, her father and brother were all positive for the ( – – ^SEA^/) deletion, but the signal for her mother was weak and inconclusive. These results were subsequently confirmed by gap‐PCR tests, documenting that the proband, her father and brother, but not her mother, were heterozygotes for the ( – – ^SEA^/) deletion.

## 5 DISCUSSION

Unless documented to be iron deficient, these carriers ought not be given iron supplement. Prolonged and unnecessary iron supplement can lead to harmful iron overload in various organs including the liver and the heart. Furthermore, family counseling is recommended to alert these carriers of the potential risk of begetting offspring with HbH disease or the devastating Hb Barts hydrops fetalis syndrome..^4^, ^6^

The thalassemia syndromes are increasingly important in global health. With emigration of populations over many decades and inter‐marriages, thalassemias are now found in all parts of the world, and not confined only in regions where malaria was and might still be endemic.

## 6 CONCLUSION

This case report highlights the importance for health care providers in all parts of the world to be aware of the alpha‐thalassemia syndromes, their relevance to clinical care and family counseling, appropriate diagnostic algorithm for definitive diagnosis,^3^ and distinction from iron deficiency.

## CONFLICT OF INTEREST

The authors declare no conflicts of interest.

## AUTHOR CONTRIBUTIONS

MS and WR: wrote the manuscript and contributed to laboratory tests and clinical follow‐up; DC: revised the manuscript and contributed to laboratory tests.

## ETHICAL APPROVAL

The study has been approved by the appropriate ethics. Published with written consent of the patients.

## Data Availability

The data that support the findings of this study are available on request from the corresponding author. The data are not publicly available due to privacy or ethical restrictions.
